# Enhanced plant diversity reduces nitrous oxide emissions in forest soils worldwide

**DOI:** 10.1093/nsr/nwaf186

**Published:** 2025-05-13

**Authors:** Hanling Zuo, Wensheng Xiao, Mingqiu Dong, Xinyun Gu, Xia Liang, Pete Smith, Manuel Delgado Baquerizo, Lijun Hou, Xiaoqi Zhou

**Affiliations:** Zhejiang Tiantong Forest Ecosystem National Observation and Research Station, Zhejiang Zhoushan Island Ecosystem Observation and Research Station, Institute of Eco-Chongming, School of Ecological and Environmental Sciences, East China Normal University, Shanghai 200241, China; Zhejiang Tiantong Forest Ecosystem National Observation and Research Station, Zhejiang Zhoushan Island Ecosystem Observation and Research Station, Institute of Eco-Chongming, School of Ecological and Environmental Sciences, East China Normal University, Shanghai 200241, China; Zhejiang Tiantong Forest Ecosystem National Observation and Research Station, Zhejiang Zhoushan Island Ecosystem Observation and Research Station, Institute of Eco-Chongming, School of Ecological and Environmental Sciences, East China Normal University, Shanghai 200241, China; Zhejiang Tiantong Forest Ecosystem National Observation and Research Station, Zhejiang Zhoushan Island Ecosystem Observation and Research Station, Institute of Eco-Chongming, School of Ecological and Environmental Sciences, East China Normal University, Shanghai 200241, China; State Key Laboratory of Estuarine and Coastal Research, East China Normal University, Shanghai 200241, China; Institute of Biological and Environmental Sciences, University of Aberdeen, Aberdeen AB24 3FX, UK; Laboratorio de Biodiversidad y Funcionamiento Ecosistémico, Instituto de Recursos Naturales y Agrobiología de Sevilla (IRNAS), Consejo Superior de Investigaciones Científicas (CSIC), Sevilla E-41012, Spain; State Key Laboratory of Estuarine and Coastal Research, East China Normal University, Shanghai 200241, China; Zhejiang Tiantong Forest Ecosystem National Observation and Research Station, Zhejiang Zhoushan Island Ecosystem Observation and Research Station, Institute of Eco-Chongming, School of Ecological and Environmental Sciences, East China Normal University, Shanghai 200241, China

**Keywords:** tree diversity, nitrous oxide, forest ecosystem, natural abundance N_2_O isotopes, model simulation

## Abstract

Forests are recognized as the largest natural source of nitrous oxide (N_2_O) emissions on land, with deforestation drastically reducing the cover and biodiversity of native forests worldwide. Yet, how losses in forest biodiversity affect soil N_2_O fluxes remains poorly understood. Here, we combined a global tree diversity–forest soil N_2_O data set, including 201 paired comparable observations from global forests, with a three-year field survey of *in-situ* flux data gathered from a long-term plant diversity field experiment. Our analyses reveal that tree diversity has a significant negative effect on soil N_2_O emissions, primarily driven by a decrease in N_2_O production associated with denitrification. More specifically, we showed that reductions in N_2_O emissions with tree diversity can be attributed to a decrease in the availability of soil inorganic nitrogen. Predictive modeling further shows that compared to forests with a single tree species, forests with two tree species can reduce global forest N_2_O emissions by 10.39%, while those with 24 tree species achieve the maximum mitigation effect, reducing emissions by 56.30%. Taken together, our work highlights the contribution of tree diversity for mitigating N_2_O emissions, highlighting the importance of accounting for biodiversity when reforesting old forests and supporting new afforestation processes.

## Teaser

Global dataset combined with field experiment demonstrates that preserving high forest plant diversity effectively mitigates N_2_O emissions, thereby contributing to global warming mitigation.

## INTRODUCTION

Deforestation is reducing the cover of native forests worldwide, with almost 10 million hectares being altered every year [1]. The rapid decline in forest biodiversity has sparked widespread concern [2,3]. Numerous studies have shown that losses in plant diversity can negatively impact aboveground biomass and ecosystem productivity [4,5], negatively impacting the biogeochemical cycles of carbon and nitrogen, the main precursors of greenhouse gas emissions [6–8]. Nitrous oxide (N_2_O) is a potent greenhouse gas, with a global warming potential 298 times that of carbon dioxide (CO_2_) on a per molecule basis over a 100-year timeframe [9]. Since the industrial revolution, the concentration of N_2_O in the atmosphere has increased by 20%, rising at a rate of 0.2% to 0.3% per year [9,10]. Forests, as the largest natural source of N_2_O emissions in terrestrial ecosystems, contribute ∼4.28 Tg N yr^−1^ of N_2_O emissions, accounting for over 60% of natural N_2_O emissions. The variations in N_2_O emissions from forests could significantly impact climate warming [10,11]. However, there is still considerable uncertainty in the current estimates of N_2_O emissions from forests [11]. A key factor contributing to this uncertainty is the insufficient consideration given to how plant diversity influences N_2_O emissions. An assessment of the contribution of forest biodiversity in driving N_2_O emissions is urgently needed to reduce the current level of uncertainty.

Current uncertainty about the role of forest biodiversity in shaping N_2_O emissions exists for three main reasons. First, studies examining the impact of plant diversity on soil N_2_O fluxes primarily focus on grassland and wetland ecosystems and are based on short-term observational studies [12,13]. Forest ecosystems as crucial areas for the global carbon and nitrogen cycles [7], the long-term implications of plant diversity on N_2_O emissions in forest ecosystems, and their quantification, have received minimal attention, and there is a particular lack of comprehensive global-scale studies in forest ecosystems. Second, we lack a conceptual framework to understand why plant diversity in forests is key for N_2_O emissions. Previous research suggests that ecosystem productivity and aboveground biomass usually increases with increasing plant diversity [14,15], leading to greater inputs of organic carbon and nitrogen into the soil [16]. This is especially important in forests capable of injecting a large amount of litter and rhizodeposition into the soil system [16]. This not only provides a carbon source for microorganisms but also supplies substrates required for N_2_O production processes [12]. Finally, forest soil N_2_O primarily originates from microbe-mediated nitrification and denitrification processes [17]. Unfortunately, it is unclear how the production of these microbial processes varies under different plant diversities, which hinders the accurate simulation of N_2_O emissions in the soil [18,19]. Current methods to quantify these processes rely heavily on cultivation experiments. For instance, 0.01% and 10% concentrations of acetylene (C_2_H_2_) can inhibit specific N_2_O production pathways, facilitating the distinction between these processes [20]. By adding inorganic nitrogen substrates labeled with the ^15^N isotope, which are required for nitrification and denitrification processes, different N_2_O production pathways can be identified [21]. However, these methods do not accurately characterize the *in-situ* microbial processes of N_2_O emissions. A novel approach based on the natural abundance of N_2_O isotopes composition was used to distinguish the *in-situ* N_2_O production contributions. This method does not require the addition of tracers, causes minimal disturbance to the soil system, and is suitable for field studies [22,23]. Consequently, it enables the tracing of soil N_2_O production and provides a more precise quantification of N_2_O emissions.

Here, we investigate the impact of tree diversity on soil N_2_O fluxes across global forests. To such an end, we combined a global data set, including information on tree diversity and soil N_2_O emissions across 201 paired comparable observations from global forests, with a three-year field study of *in-situ* flux data from a long-term plant diversity field experiment. By measuring the natural abundance of N_2_O isotopes we were able to differentiate and quantify the sources of soil N_2_O flux. Finally, we incorporated tree diversity factors into a process-based model to estimate global forest N_2_O emissions under different tree diversity scenarios. This study aimed to elucidate the significant role of changes in tree diversity in global forest soil N_2_O emissions.

## RESULTS

### The contribution of tree diversity as a regulator of forest soil N_2_O fluxes

**Figure 1.**
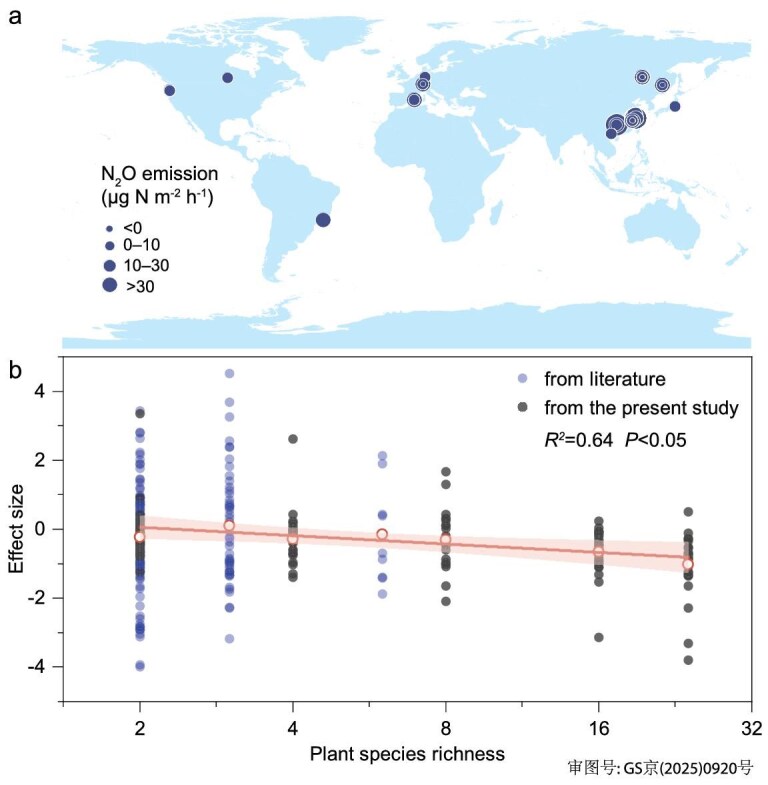
(a) Distribution of sampling points in the global tree diversity–forest soil N_2_O flux data set constructed from the meta-analysis and field observation data. (b) Linear relationship between tree diversity and the effect size of tree diversity on N_2_O flux. N_2_O flux is composed of meta-analysis data and three years of field observation data. The hollow circles represent the average N_2_O flux under different plant diversities. The line indicates the weighted linear fit to the mean values and standard error, with the shaded area indicating the 95% confidence interval of the linear fit. A Log2 transformation has been applied to the x-axis.

Our analyses indicate that losses in tree diversity can have critical consequences for N_2_O emission across forests worldwide. First, by combining a global forest data set with an *in-situ* experiment, we found that soil N_2_O emission rates in global forests ranged from −10.13 to 121.14 μg m^−2^ h^−1^, with a median of 5.29 (95% CI, −3.52 to 38.81) μg m^−2^ h^−1^. To ensure data comparability, we calculated effect sizes to assess the effects of tree diversity on soil N_2_O emissions (Fig. 1, Fig. S2). Tree diversity had a significant negative effect on soil N_2_O fluxes across forests (*R^2^*= 0.64, *P* < 0.05) (Fig. 1). We calculated the effect sizes of tree diversity on N_2_O fluxes using both the literature data set and the biodiversityecosystem functioning experiment China (BEF-China) experimental data set, and conducted subgroup analyses (Fig. S2). Overall, in the literature data set, the effect size of total tree species mixtures on forest N_2_O fluxes was negative (*P* < 0.05). The effect sizes for mixtures containing 2 and 6 tree species were significantly different from zero (*P* < 0.05), whereas the effect size for mixtures with 3 tree species was not statistically significant (Fig. S2). Similarly, in the BEF-China experimental data set, the effect size of total species mixtures on forest N_2_O fluxes was negative (*P* < 0.001), with a notable decrease in effect size as tree diversity increased (Fig. S2).

**Figure 2.**
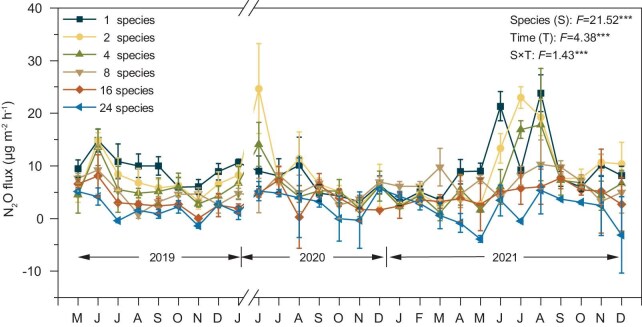
Three-year field observation experiment on the BEF-China platform to investigate the effects of different tree diversity scenarios on forest soil N_2_O fluxes. The BEF-China platform was constructed in 2009. *In-situ* forest soil N_2_O fluxes at 6 tree diversity (1, 2, 4, 8, 16, 24) levels were observed by using the static chamber method from May 2019 to December 2021. The x-axis is time from May 2019 to December 2021. Values are means ± standard errors. Significance levels at *P* < 0.05, *P* < 0.01, *P* < 0.001 are indicated by *, **, ***, respectively.

**Figure 3.**
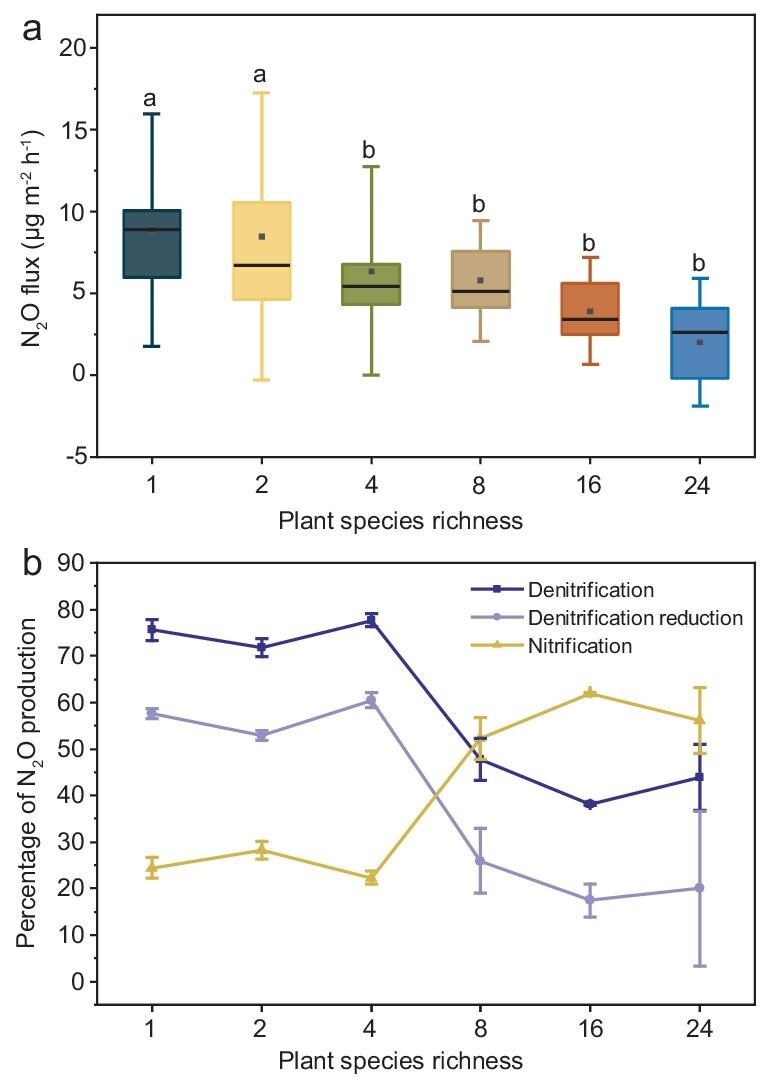
(a) The average emission rate of subtropical forests’ *in-situ* soil N_2_O under different tree diversities. (b) Percentages of N_2_O production or consumption in the nitrification process, denitrification process and denitrification reduction process. The box represents the 25th, median and 75th percentiles, while the whiskers indicate the standard deviation. The small black squares represent the mean values, with different lowercase letters indicating significant differences in the mean values.

Our *in-situ* experiment further demonstrated significant differences in forest soil N_2_O emissions under different tree diversities (*P* < 0.001) (Fig. 2, Table S2). We further found an interaction effect between tree diversity and sampling time (Fig. 2), with N_2_O emissions during the growing season (April to October) being higher than those in the non-growing season (November to March) (Fig. S3). By measuring the natural abundance of N_2_O isotopes, we found significant changes in the isotope values of 8, 16 and 24 tree species compared to 1, 2 and 4 tree species (Fig. 3, Fig. S4). We further found that, under low tree diversity, bacterial denitrification dominated soil N_2_O production in forest ecosystems. As tree diversity increases, the proportion of N_2_O production from bacterial denitrification decreases, while the proportion from nitrification increases (Fig. 3).

### Direct and indirect effects of tree diversity on N_2_O emissions

**Figure 4.**
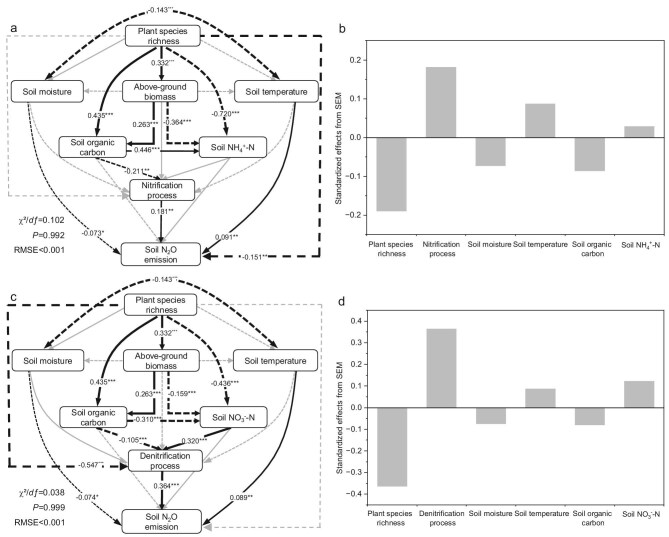
Structural equation model describing the effects of tree diversity, soil moisture, soil temperature, soil organic matter and soil inorganic nitrogen on *in-situ* soil N_2_O emissions from (a) nitrification processes and (c) denitrification processes in a subtropical forest. (b, d) Standardized total effects (direct plus indirect effects) derived from structural equation modeling were also calculated. Black arrows indicate significant and gray arrows indicate non-significant. Continuous and dashed arrows indicate positive and negative relationships, respectively. Arrow width is proportional to the strength of the relationship. Significance levels at *P* < 0.05, *P* < 0.01, *P* < 0.001 are indicated by *, **, ***, respectively.

We constructed structural equation models (SEMs) to further investigate the nitrification and denitrification processes, respectively, while accounting for the multiple direct and indirect environmental factors influencing these microbe-driven processes (Fig. 4). We quantified the indirect effect of tree diversity on soil N_2_O fluxes via changes in aboveground biomass, soil temperature, soil moisture, soil organic carbon and soil inorganic nitrogen (Fig. 4). The results showed that the contribution of the denitrification process to N_2_O flux was higher than that of the nitrification process. Tree diversity showed a direct positive correlation with aboveground biomass and soil organic carbon accumulation, but reduced soil inorganic nitrogen contents (Fig. 4, Fig. S5). In the denitrification process, the reduction of soil nitrate nitrogen (NO_3_^−^-N) significantly reduced the denitrification N_2_O production, while the effect between soil ammonium nitrogen (NH_4_^+^-N) and the nitrification process was not significant (Fig. 4). The increase of soil organic carbon promoted the accumulation of soil NH_4_^+^-N, but decreased the content of soil NO_3_^−^-N, and soil organic carbon had a negative effect on both nitrification and denitrification processes (Fig. 4). In addition, we found that tree diversity had no significant effect on soil moisture and soil temperature (Fig. S6). However, both soil temperature and soil moisture had a direct effect on N_2_O fluxes (Fig. 4, Fig. S5). Notably, there were differences in soil temperature between the growing season and the non-growing season (Fig. S6).

### Estimating global forest N_2_O emissions under different tree diversity scenarios

**Figure 5.**
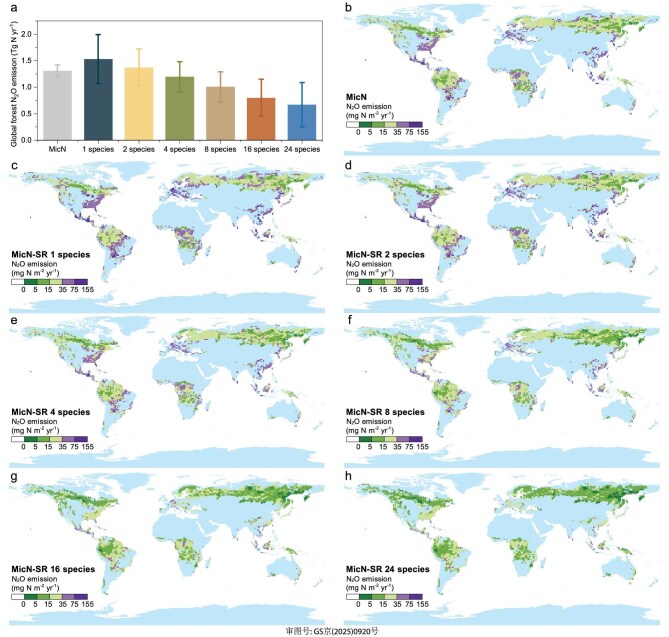
(a) Model simulation of global forest soil N_2_O emissions under different tree diversity scenarios. Spatial distribution patterns of global forest soil N_2_O emissions: (b) MicN-model-simulated, and MicN-SR-model-simulated (c) single species, (d) 2 species, (e) 4 species, (f) 8 species, (g) 16 species and (h) 24 species.

We then integrated tree diversity factors into a process-based N_2_O emission Microbial Nitrogen model (MicN) [24], and a plant species richness–soil-N_2_O flux model (MicN-SR) to quantify global forest soil N_2_O fluxes under different tree diversity scenarios (Fig. 5). Compared to the MicN model, the MicN-SR model demonstrates better validation results in forests worldwide (*R^2^*= 0.49) (Fig. S7). After incorporating diversity factors, global forest soil N_2_O emissions were estimated to range from 0.67 ± 0.42 to 1.53 ± 0.46 Tg N yr^−1^. In comparison to a single tree species, soil N_2_O emissions at the 16 and 24 tree species levels respectively decreased by 47.87% and 56.30% (Fig. 5). After distinguishing forest types based on climate zones, it was found that tropical/subtropical forests contribute to ∼60% of global forest N_2_O emissions (Fig. S8).

## DISCUSSION

### Tree diversity explains forest soil N_2_O emissions

Our findings demonstrate that an increase in tree diversity can play a role in the mitigation of global forest soil N_2_O emissions (Fig. 1). We demonstrated this by combining a global data set of forest tree diversity and N_2_O emissions with a three-year forest experiment. Based on *in-situ* natural abundance isotopes of N_2_O, we found an increase in tree diversity and a decrease in N_2_O emissions (Fig. 2, Table S2), which was mainly due to decreased N_2_O production from nitrification and denitrification processes (Fig. 3). Notably, the decrease in denitrification is more significant, making it the dominant process affecting N_2_O flux changes (Fig. 3). We also divided the annual fluxes into growing and non-growing seasons, yielding similar results and higher N_2_O emissions during the growing season (Fig. S3).

We then used structural equation modeling aiming to identify the mechanisms through which tree diversity influences N_2_O production processes (Fig. 4). First, tree diversity reduced soil inorganic nitrogen contents (NH_4_^+^-N and NO_3_^−^-N), which are direct substrates for nitrification and denitrification of N_2_O production [25,26], by increasing aboveground biomass as an intermediary variable (Fig. 4, Fig. S5). The connection between tree diversity, aboveground biomass and soil inorganic nitrogen contents can be explained by the complementarity effect [27]. Previous studies based on the same sites have demonstrated a significant positive correlation between tree diversity and complementarity effect [28]. Complementarity effect enables different tree species to occupy a greater number of ecological niches, allowing for a more effective use of the limited nutrient resources within the ecosystem [29,30]. Plants utilize more soil inorganic nitrogen for their growth [14,29], resulting in nitrogen competition with microbes [31,32], and reduce the soil inorganic nitrogen required by nitrifying and denitrifying microbes, thereby decreasing N_2_O production in the soil.

Second, soil organic carbon acts as a crucial energy and carbon source for microbial activity [29], and tree diversity increases both the quantity and quality of root exudates and leaf litter [33,34], leading to enhanced organic carbon inputs into the soil (Fig. 4, Fig. S5). However, we observed that soil organic carbon negatively impacts the nitrification and denitrification processes (Fig. 4). Higher plant diversity creates additional space and ecological niches, thereby enhancing the biomass and diversity of soil microorganisms [34,35]. These microbial communities compete with nitrifying and denitrifying microbes for energy, carbon sources and nutrients, thus limiting the survival of nitrifying and denitrifying microorganisms, directly influencing rates of soil carbon and nitrogen cycling [36]. Furthermore, previous studies have suggested that increased plant species richness may reduce the mineralization rates of both soil organic carbon and nitrogen, as well as the efficiency of microbial utilization of soil inorganic nitrogen [8,35,37]. This reduction can subsequently affect N_2_O production during nitrification and denitrification processes.

Finally, tree diversity can alter the environmental conditions necessary for microbial N_2_O production. For instance, denitrification typically occurs in moist anaerobic environments [38]. Previous studies have shown that increased plant diversity intensifies competition among plants for soil moisture, thereby affecting the anaerobic conditions required for denitrification [39]. However, this study found no direct relationship between tree diversity and soil moisture (Fig. 4, Fig. S6). Nevertheless, under high plant diversity, the root structures of plants become more complex, enhancing soil aeration and subsequently decreasing denitrification N_2_O production [13,40,41]. It is noteworthy that there are no significant differences in soil moisture between the growing and non-growing season, whereas soil temperatures during the growing season are higher than those in the non-growing season. Forest N_2_O fluxes were elevated in the growing season compared to the non-growing season, likely due to the increased soil temperatures that stimulate microbial activity [42].

### Simulation of global forest soil N_2_O fluxes under different tree diversity scenarios

Typically, process-based models, statistical extrapolation, or remote sensing atmospheric inversion methods are employed to estimate natural soil N_2_O emissions [42]. However, these methods have significant uncertainties in estimating N_2_O fluxes. For example, process-based models are limited by observational data and do not incorporate the complex microbial processes involved [43]. Furthermore, the complexity and high heterogeneity of forests make it difficult to conduct biodiversity control experiments to obtain *in-situ* N_2_O observations under different plant diversities [42,44]. Current process models have not taken into consideration the influence of changes in plant diversity on N_2_O fluxes [45]. This study has identified a significant potential for reducing forest soil N_2_O emissions by increasing tree diversity (Fig. 1). Based on this, a process-based model [24], and incorporation of a tree diversity factor, were used to build a MicN-SR model and to quantify the N_2_O emission reduction effects of increasing tree diversity.

The simulation results from the MicN-SR model show a significant decrease in global forest soil N_2_O emissions as tree diversity increases (Fig. 5). Compared to a single tree species, having 8, 16 and 24 tree species leads to N_2_O emission reductions of 34.33%, 47.87% and 56.30%, respectively. Under two tree species scenarios, the global forest N_2_O emissions were similar to the simulation results of MicN model (Fig. 5), and this result falls within the range of global forest N_2_O emissions estimated by previous studies [43]. The spatial distribution patterns of N_2_O emissions observed in this study are generally consistent with the results of other studies [10,11]. About 60% of this N_2_O emission reduction occurs in tropical/subtropical forests (Fig. S8). Compared to temperate/boreal forests, tropical/subtropical forests have a greater coverage area, higher temperatures and relatively enriched nitrogen content in their soils [46]. These factors lead to a higher nitrogen turnover rate in these soils, resulting in N_2_O emissions from tropical/subtropical forest soils that exceed those from temperate/boreal forests [46,47]. Additionally, the high moisture saturation levels in tropical and subtropical forest regions create anaerobic environments, along with the relative enrichment of NO_3_^−^-N [38], which facilitates N_2_O production primarily driven by the denitrification process.

Quantifying N_2_O fluxes in large-scale terrestrial ecosystems using *in-situ* methods heavily relies on long term N_2_O flux observation data, which are often unavailable [42,43]. The process models can enhance the comprehensiveness of estimations by integrating various data sources, such as meteorological and soil data [24]. However, these models face challenges, including obtaining accurate parameter estimates [42,48,49]. In this study, we combined long-term *in-situ* N_2_O flux observations with global forest N_2_O flux observations from multiple locations to construct the MicN-SR model, and for the first time quantified the impact of tree diversity on soil N_2_O emissions in global forests. Global validation results indicate that the model explanatory power improved by 22% (Fig. S7). Our research underscores the importance of considering tree diversity when studying forest ecosystem N_2_O emissions. However, the MicN-SR model still has limitations due to a lack of data on N_2_O emissions from boreal and tropical forest soils under high tree diversity scenarios. The validation of the simulation results under high tree diversity was mainly based on subtropical forests. Future research should conduct monitoring experiments of forest biodiversity and N_2_O fluxes in different regions to obtain field observation data and further improve the accuracy of predictions.

A wealth of research has established a positive relationship between biodiversity and productivity, as well as ecosystem functions, which can significantly alter ecosystem carbon and nitrogen cycling, thereby impacting greenhouse gas emissions [7,12,16]. Our study further elucidates the critical relationship between tree diversity and forest N_2_O emissions, offering significant guidance for sustainable forest development in the context of climate change. This research aligns with the goals of the United Nations Strategic Plan for Forests 2017–2030, which aims to increase global forest area by 3% by 2030. Therefore, tree diversity factors should be considered in future production practices and forest management strategies. Approaches such as afforestation and mixed-species plantings can enhance the overall resilience of ecosystems to climate change, ultimately achieving the dual goals of scientific conservation and management of forest ecosystems and mitigating global climate change.

## CONCLUSION

Our work, supported by a comprehensive global data set and three years of *in-situ* forest experiments, provides evidence that tree diversity is a major regulator of N_2_O fluxes in forests. The findings indicate that an increase in tree diversity leads to a reduction in soil N_2_O emissions. We identified the sources of N_2_O flux and found that the decrease in N_2_O under high tree diversity levels mainly results from the bacterial denitrification process. Higher tree diversity diminishes the availability of soil inorganic nitrogen required for the bacterial denitrification process, thereby lowering N_2_O production from these processes. Predictive modeling further shows that compared to forests with a single tree species, forests with two tree species can reduce global forest N_2_O emissions by 10.39%, while those with 24 tree species achieve the maximum mitigation effect, reducing emissions by 56.30%. This study first quantifies the potential of increasing tree diversity in reducing forest soil N_2_O emissions, highlighting the importance of maintaining high tree diversity in forests as a strategy to mitigate the impacts of global climate change. We recommend incorporating tree diversity into terrestrial ecosystem N_2_O flux models to better quantify the dynamic changes in forest N_2_O under future climate scenarios.

## MATERIALS AND METHODS

### Constructed global tree diversity–forest soil N_2_O flux data set

To assess the general effect of tree diversity on soil N_2_O fluxes in global forests, we constructed a global tree diversity–forest soil N_2_O flux data set, including paired observations from published studies, with three years of N_2_O fluxes from a long-term plant diversity field experiment. We retrieved relevant research articles before April 2024 from the Web of Science (https://www.webofscience.com), using the keywords [N_2_O OR Nitrous Oxide] and [Forest]. The articles were chosen based on specific criteria: (i) the utilization of automatic or manual static chamber methods for gas flux monitoring; (ii) soil N_2_O flux data that could be directly extracted from text, tables and figures; (iii) accurate information on tree species richness, along with a comparison of soil N_2_O emissions from single-tree-species forests and mixture-of-tree-species forests. For each study, data on latitude, longitude, temperature, rainfall, duration of observation, N_2_O flux, number of replicates, standard error, species richness and species composition were collected for each site, and data were extracted from images using GetData (see supplementary data: Additional information 1). Paired comparable observations from global forests (201 in total) include: boreal forest (58), temperate forest (75), subtropical forest (17) and tropical forest (51).

The effect size of tree diversity on soil N_2_O fluxes was quantified by calculating the standardized mean difference (SMD) [50] and conducting a subgroup analysis of the effect size of tree diversity on forest N_2_O fluxes. Each article provided a single-tree-species plot that served as the control group, while a mixture-of-tree-species sample plot served as the treatment groups.


(1) \begin{eqnarray*}
{\rm SMD} = ({X}_T - {X}_C)/S{D}_{cob}.
\end{eqnarray*}

SMD is the effect size, ${X}_C$ and$\ {X}_T$ are the means of the control groups (single tree species) and the treatment groups (mixture of tree species), and $S{D}_{cob}$ is the combined standard deviation of the two groups.

### Observations of *in-situ* forest soil N_2_O flux under different tree diversity

The *in-situ* observation experiment of this study was conducted at the Biodiversity–Ecosystem Functioning Experiment China (www.bef-china.com) in Jiangxi Province, China (29.13°N, 117.91°E). The climate belongs to the subtropical monsoon category. Site A of this experimental platform was selected for the study [51] (Fig. S1). Detailed information regarding the plot design can be found in Bruelheide *et al.* (2014) [44] and supplementary data: Additional information 2. In this study, we selected 6 tree diversity levels: single species (16 plots), 2 species (8 plots), 4 species (4 plots), 8 species (2 plots), 16 species (1 plot) and 24 species (1 plot) (Table S1). The sampling period was from May 2019 to December 2021. Soil N_2_O flux observations were conducted *in situ* under different tree diversity levels using the static chamber method [52]. A syringe was pushed and pulled 4–5 times to ensure uniform mixing of the gas within the chamber. Gas samples were then collected at 0, 10, 20, 30 and 40 minutes, respectively, and injected into 10 mL glass vial tubes and transported to the laboratory, where they were analyzed using a gas chromatograph (Agilent 7890B GC, USA) within 48 hours. The N_2_O flux was calculated using the following formula [53]:


(2) \begin{eqnarray*}
f = \frac{{\Delta m}}{{A \times \Delta t}} = \frac{{\rho v\Delta c}}{{A \times \Delta t}} = \rho h \times \frac{{\Delta c}}{{\Delta t}}.
\end{eqnarray*}

In this equation, *f* represents the N_2_O flux (μg m^−2^ h^−1^); $\frac{{\Delta m}}{{\Delta t}}$ indicates the change in N_2_O mass inside the chamber over time $\Delta t$; $\frac{{\Delta c}}{{\Delta t}}$ signifies the change in N_2_O concentration inside the chamber over time $\Delta t$; $\rho $ denotes the density of N_2_O at the chamber temperature; and *A, v* and *h* correspond to the base area, volume and height of the static chamber, respectively.

### Measurements of the natural abundance of *in-situ* N_2_O isotopes and quantification of N_2_O production

By measuring the natural abundance of *in-situ* N_2_O isotopes (δ^15^N^SP^_N_2_O_, δ^18^O_N_2_O_ and δ^15^N^bulk^_N_2_O_), in combination with isotope mapping (SP/O MAP) [22,23,54], we identified and quantified the sources of N_2_O flux. The isotopic ratios of N_2_O (δ^15^N^bulk^_N_2_O_, δ^15^N^α^ and δ^18^O_N_2_O_) were analyzed using a gas chromatograph-isotope ratio mass spectrometer (GC-IRMS, Thermo Fisher Scientific [China] Co., Ltd., Beijing, China) [25]. δ^15^N^SP^_N_2_O_ represents the difference in ^15^N/^14^N between the central N atom of N_2_O (δ^15^N^α^) and the terminal nitrogen atom (δ^15^N^β^), calculated using the following formula [55]:


(3) \begin{eqnarray*}
{\delta }^{15}{\rm N}^{\rm SP}_{{\rm N}_2{\rm O}} = {\delta }^{15}{\rm N}^\alpha - {\delta }^{15}{\rm N}^\beta.
\end{eqnarray*}

(4) \begin{eqnarray*}
{\delta }^{15}{\rm N}^{{\rm bulk}}_{{\rm N}_2{\rm O}} = ({\delta }^{15}{\rm N}^\alpha + {\delta }^{15}{\rm N}^\beta )/2.
\end{eqnarray*}

The proportions of nitrification (Ni), bacterial denitrification (bD) and N_2_O reduction processes contributing to N_2_O production or consumption were quantified using the SP/O MAP model. The initial parameters in the SP/O MAP were adjusted according to the actual ^18^O_H_2_O_ isotopes present in the soil. The δ^15^N^SP^_N_2_O_ values are unaffected by the isotopic composition of the substrates [56]. Details on the parameters of the SP/O MAP model can be found in Table S3. This calculation method is consistent with those previously published by our team [26], with specific computational details found in the Additional information 2.

### Soil sample collection and measurement of the soil physicochemical properties

The five-point sampling method was employed to collect soil samples at depths of 0–10 cm from each plot. The collected samples were stored in a cooler and transported to the laboratory for processing [57]. Initially, the soil samples underwent sieving (2 mm mesh) to remove stones and roots, and were then stored in a refrigerator at 4°C for subsequent analysis [58]. Some of the soil samples were dried, ground and analyzed for total carbon and total nitrogen using an elemental analyzer (Vario MICRO cube, Elementar, Germany). The study area is a typical southern acidic soil (pH: 4.5–5.5), with virtually no inorganic carbon content. Therefore, the total carbon content in the soil is equivalent to the organic carbon content [58]. The concentrations of soil ammonium nitrogen (NH_4_^+^-N) and nitrate nitrogen (NO_3_^−^-N) were analyzed using an automated discrete chemical analyzer (Smartchem 200 AMS, Italy). Soil moisture and pH measurements were conducted following the methods outlined in Gu *et al.* (2019) [57]. To eliminate the influence of slope on the results, we calculated the slope factor (soil nutrient loss rate) for each plot [59], and adjusted the *in-situ* fluxes, isotope data and soil physicochemical data. The calculation of the slope factor is as follows:


(5) \begin{eqnarray*}
S = {(\theta /10)}^{0.78},
\end{eqnarray*}

where *S* represents the slope factor and $\theta $ represents the slope (°).

### Simulating global forest soil N_2_O fluxes under different tree diversity scenarios

Based on the MicN model [24], we constructed the MicN-SR model, which includes four processes associated with N_2_O production and consumption: autotrophic nitrification, heterotrophic nitrification, nitrifying bacteria denitrification and denitrifying bacteria denitrification [24]. In order to evaluate the influence of tree diversity on forest soil N_2_O emissions, we have utilized *in-situ* observation data from 2019 to 2020. By leveraging the Bayesian optimization program within the R language package ‘mlrMBO’, we have determined the maximum oxidation/reduction rates (${K}_{max\_Nitr\_AOA}$, ${K}_{max\_Nitr\_AOB}$, ${K}_{max\_Nitr\_NOB}$, ${K}_{max\_NitrH}$, ${K}_{max\_N{O}_X\_Denitr}$, ${K}_{max\_N{O}_X\_AOB}$) of nitrifying/denitrifying microbes under different tree diversity scenarios, thereby establishing the relationship between tree diversity and ${K}_{max}$, defined as the tree diversity factor (${F}_{PD}$) (see supplementary data: Additional information 2).

We divided the global tree diversity–forest soil N_2_O data set, comprising 201 paired comparable observations from global forests and three years of *in-situ* flux data from a long-term plant diversity field experiment, into training data sets (70%) and validation data sets (30%). Using the training data set to constrain the model, we employed Bayesian optimization programs in the ‘mlrMBO’ package in R language to derive MicN model parameters suitable for global-scale applications. This optimization procedure underwent 1000 iterations, and the average of the final 100 runs was chosen as the model parameters (see supplementary data: Additional information 2). Under these parameter conditions, and incorporating tree diversity factor (${F}_{PD}$), we used the MicN-SR model to calculate monthly variations in global forest ecosystem soil N_2_O emissions at a resolution of 1° × 1° under 1, 2, 4, 8, 16 and 24 species. Subsequently, we assessed the annual soil N_2_O emissions for each grid cell by applying regional weighting calculations. Finally, we used the validation data set to evaluate the MicN-SR model's simulations of global forest N_2_O fluxes under different tree diversity scenarios and calculated the model's coefficient of determination (*R^2^*) and root mean squared error (RMSE) to assess its accuracy by comparing the predictive performance of MicN-SR and MicN.

### Statistical analysis

To ensure equal spacing on the X-axis, we applied a Log2 transformation on plant species richness before conducting linear regression analysis to explore the link between tree diversity and soil N_2_O fluxes. Differences among various tree diversity levels were evaluated using ANOVA analysis with significance set at *P* < 0.05. We applied the non-parametric Kruskal-Wallis test to the data, which did not meet the normality assumption. For investigating the mechanisms by which tree diversity influences soil N_2_O flux in forest ecosystems, we initially calculated correlation coefficients between soil N_2_O fluxes and factors such as tree diversity, nitrification N_2_O production, denitrification N_2_O production, denitrification N_2_O reduction, above-ground biomass, soil temperature, soil moisture, soil organic carbon, NH_4_^+^-N and NO_3_^−^-N. Prior to conducting Pearson correlation analysis, we performed a normal transformation on the data. Subsequently, structural equation modeling was employed to analyze the key drivers impacting soil N_2_O fluxes. The structural equation model was constructed using the ‘lavvan’ package. All statistical analyses and modeling procedures in this study were performed using R 4.3.0.

## Supplementary Material

nwaf186_Supplemental_File

## Data Availability

Original data and model code can be accessed on the website https://doi.org/10.6084/m9.figshare.26319067. Soil temperature data were obtained from CRU TSv4.05 at https://crudata.uea.ac.uk/cru/data//hrg/cru_ts_4.05/ (version 4.05, variable tmp, file cru_ts4.05.2011.2020.tmp.dat.nc.gz); soil moisture data were obtained from GLEAMv3.6b at https://www.gleam.eu/ (variable Surface Soil Moisture); soil pH, soil total C, soil total N data were obtained from http://globalchange.bnu.edu.cn/research/soilw (files TC1, TN1 and PHH2O1); and soil NH_4_^+^ and NO_3_^−^ data were obtained from http://doi.org/10.22033/ESGF/CMIP6.8255. The vegetation mask was obtained from https://sage.nelson.wisc.edu/data-and-models/datasets/ (file Global Potential Vegetation Dataset).
