# Supplementary material for: Enhanced plant diversity reduces nitrous oxide emissions in forest soils worldwide
Source: Natl Sci Rev. 2025 May 13;12(7):nwaf186. doi: 10.1093/nsr/nwaf186 (PMC12168753; doi:10.1093/nsr/nwaf186)
Supplement: nwaf186_Supplemental_File [file nwaf186_supplemental_file.zip › 3. Additional information2.docx]

**Additional information 2**

**Enhanced plant diversity reduces nitrous oxide emissions in forest soils worldwide**

**1. Observations *in-situ* forests soil N_2_O flux under different tree diversity**

The *in-situ* experiment of this study was conducted at Site A of the Biodiversity-Ecosystem Function Experiment Research Platform, China. Situated at an elevation of 105-275 meters, Site A covers an area of approximately 18.4 hectares and comprises 271 basic plot units, each measuring 25.82 meters by 25.82 meters. Within each plot unit, 400 seedlings were planted with a spacing of 1.29 meters between individual plants. Following a biodiversity experimental design, tree species were randomly selected from a species pool of 24 plants to establish experimental communities, with 1, 2, 4, 8, 16, and 24 tree species included. Twice a year, shrubs and herbaceous plants in the plot were cleared to preserve the original species composition of the site. Detailed information of experimental design can be referred to Bruelheide *et al.* (2014) [1].

**2. Measurements natural abundance of *in-situ* N_2_O isotopes and quantifying N_2_O production**

N_2_O is an asymmetric molecule with nitrogen atoms labeled as α-N and β-N at the center and end of the molecule [2,3]. During the processes of N_2_O production and reduction, the breaking of molecular bonds can result in variations in the distribution of ^15^N on the two nitrogen atoms, known as the site preference value (δ^15^N^SP^_N2O_) [4]. Microorganisms and enzymes regulate different pathways during N_2_O production, leading to fluctuations in the δ^15^N^SP^_N2O_ values, this feature is independent of the isotopic composition of the substrate, making δ^15^N^SP^_N2O_ a crucial indicator for tracing N_2_O production pathways [2]. The δ^15^N^α^, δ^15^N^β^, δ^15^N^SP^_N2O_, δ^15^N^bulk^_N2O_ and δ^18^O_N2O_, values were calculated using the methods of [3,5].

$$\begin{aligned} \delta^{15}N^{\alpha}={{}^{15}{R_{sample}^{\alpha}}}/{{}^{15}{R_{std}}}-1\#\left( 1 \right) \end{aligned}$$

$$\begin{aligned} \delta^{15}N^{\beta}={{}^{15}{R_{sample}^{\beta}}}/{{}^{15}{R_{std}}}-1\#\left( 2 \right) \end{aligned}$$

$$\begin{aligned} \delta^{18}O={{}^{18}{R_{sample}}}/{{}^{18}{R_{std}}}-1\#\left( 3 \right) \end{aligned}$$

$$\begin{aligned} \delta^{15}{N^{SP}}_{N_{2}O}=\delta^{15}N^{\alpha}-\delta^{15}N^{\beta}\#\left( 4 \right) \end{aligned}$$

$$\begin{aligned} \delta^{15}{N^{bulk}}_{N_{2}O}=\left（ \delta^{15}N^{\alpha}+\delta^{15}N^{\beta} \right）/2\#\left( 5 \right) \end{aligned}$$

The SP/O MAP isotope mixing model (δ^15^N^SP^_N2O_/δ^18^O_N2O_) can distinguish the contributions of two pathways to N_2_O emissions. For simplicity, this study primarily focuses on nitrification (Ni) and bacterial denitrification (bD) [4]. In the SP/O MAP, a mixing line and a reduction line are defined, encompassing two scenarios: 1) Reduction-Mixing: N_2_O produced through denitrification is first reduced, and the remaining N_2_O is mixed with that produced by nitrification; 2) Mixing-Reduction: N_2_O produced by both denitrification and nitrification is mixed first, after which N_2_O is reduced to N_2_. This study employed Mixing-Reduction scenario, which is more suitable for organic matter-rich soils [4,6]. Based on the isotopic values of the samples, we calculated the contributions of nitrification and bacterial denitrification to N_2_O production under different plant diversity. The initial parameters in the SP/O MAP were adjusted according to the actual oxygen isotopes present in the soil [7]. A detailed description of the methods is provided by Wu et al. (2019) [6], and the main steps are outlined as follows:

$$\begin{aligned} Y=a_{mix}\times X+b_{mix}\#\left( 6 \right) \end{aligned}$$

$$\begin{aligned} Y=a_{red}\times X+b_{red}\#\left( 7 \right) \end{aligned}$$

$$\begin{aligned} a_{mix}=\left( Y_{Ni}-Y_{bD} \right)/\left( X_{Ni}-X_{bD} \right)\#\left( 8 \right) \end{aligned}$$

$$\begin{aligned} b_{mix}=Y_{bD}-X_{bD}\times a_{mix}\#\left( 9 \right) \end{aligned}$$

$$\begin{aligned} b_{red}=Y_{sample}-X_{sample}\times a_{red}\#\left( 10 \right) \end{aligned}$$

In the equation, $Y$ and $X$ represent the δ^15^N^SP^_N2O_（$Y_{Ni}$、$Y_{bD}$）and δ^18^O_N2O_（$X_{Ni}$、$X_{bD}$）values of nitrification (Ni) and bacterial denitrification (bD); $a_{mix}$、$a_{red}$、$b_{mix}$、$b_{red}$ represent the slopes and intercepts of the mixing line and reduction line，$a_{red}$=0.33; $Y_{sample}$、$X_{sample}$ represent the values measured for the samples.

$$\begin{aligned} X_{int}=\left( b_{mix}-b_{red} \right)/\left( a_{red}-a_{mix} \right)\#\left( 11 \right) \end{aligned}$$

$$\begin{aligned} Y_{int}={a_{red}\times X_{int}+b}_{red}\#\left( 12 \right) \end{aligned}$$

$$\begin{aligned} r_{N2O}=\exp\left( {(Y_{sample}-Y_{int})}/{\eta_{red}} \right)\#\left( 13 \right) \end{aligned}$$

$$\begin{aligned} f_{bD}=\left( Y_{int}-Y_{Ni} \right)/\left( Y_{bD}-Y_{Ni} \right)\#\left( 14 \right) \end{aligned}$$

$$\begin{aligned} f_{Ni}=100\%-f_{bD}\#\left( 15 \right) \end{aligned}$$

$$\begin{aligned} r_{reduction}=\left( 1-r_{N2O} \right)\times100\#\left( 16 \right) \end{aligned}$$

In the equation, $X_{int}$、$Y_{int}$ represent the δ^15^N^SP^_N2O_ and δ^18^O_N2O_ values of the N_2_O that is mixed prior to reduction; $r_{N2O}$indicates the proportion of unreduced N_2_O; $\eta_{red}$ indicates the proportion of unreduced N_2_O; $f_{bD}$ indicates the proportion of unreduced N_2_O; $f_{Ni}$ represents the proportion produced through nitrification; $r_{reduction}$indicates the proportion of N_2_O.

**3. Construction of plant-soil-N_2_O flux model**

Based on the Microbial Nitrogen model (MicN) [8], we added plant diversity factors to the model, constructed the plant diversity-soil-N_2_O flux model (MicN-SR). Model driven data and calculation formulas are available at “Additional information 2 Table S1 and S2”, Parameter optimization results and parameter uncertainties are shown in “Additional information 2 Table S3 and S4”

**(1) Autotrophic nitrification**

Autotrophic nitrification is the process in which microbes, under aerobic conditions, convert NH_4_^+^ into NO_2_^-^ and NO_3_^-^, while also releasing N_2_O. In the initial stage of autotrophic nitrification, NH_4_^+^ is oxidized to NO_2_^-^, a process that is facilitated by both ammonia-oxidizing archaea (AOA) and ammonia-oxidizing bacteria (AOB) [9]. The rate of this reaction primarily depends on the concentration of NH_4_^+^ [10], soil temperature [11], soil moisture content [12], soil pH [13], and plant diversity. Thus, the NH_4_^+^ oxidation rates driven by AOA ($R_{Nitr\_AOA}$, g N m^-3^ h^-1^) and AOB ($R_{Nitr\_AOB}$, g N m^-3^ h^-1^) can be expressed as:

$R_{Nitr\_AOA}=K_{max\_Nitr\_AOA}\times\frac{c_{{NH}_{4}^{+}}}{{c_{{NH}_{4}^{+}}+K}_{{NH}_{4}^{+}\_Nitr\_AOA}}\times F_{t\_Nitr\_AOA}\times F_{WFPS\_Nitr\_AOA}\times F_{pH\_Nitr\_AOA}\times F_{PD\_Nitr\_AOA} (17)$

$R_{Nitr\_AOB}=K_{max\_Nitr\_AOB}\times\frac{c_{{NH}_{4}^{+}}}{{c_{{NH}_{4}^{+}}+K}_{{NH}_{4}^{+}\_Nitr\_AOB}}\times F_{t\_Nitr\_AOB}\times F_{WFPS\_Nitr\_AOB}\times F_{pH\_Nitr\_AOB}\times F_{PD\_Nitr\_AOB} (18)$

Where $K_{max\_Nitr\_AOA}$ and $K_{max\_Nitr\_AOB}$ are the maximum NH_4_^+^ oxidation rates by AOA and AOB (g N m^-3^ h^-1^); $c_{{NH}_{4}^{+}}$ is the concentration of NH_4_^+^ in soil (mg kg^-1^); $K_{{NH}_{4}^{+}\_Nitr\_AOA}$ (0.2 g N m^-3^ h^-1^) and $K_{{NH}_{4}^{+}\_Nitr\_AOB}$ (3.0 g N m^-3^ h^-1^) are the half-saturation coefficients of NH_4_^+^ for AOA and AOB; $F_{t\_Nitr\_AOA}$ and $F_{t\_Nitr\_AOB}$ represent the restriction of soil temperature on AOA and AOB; $F_{WFPS\_Nitr\_AOA}$ and $F_{WFPS\_Nitr\_AOB}$ represent the restriction of soil moisture on AOA and AOB; $F_{pH\_Nitr\_AOA}$ and $F_{pH\_Nitr\_AOB}$ represent the restrictions of soil pH on AOA and AOB; $F_{PD\_Nitr\_AOA}$ and $F_{PD\_Nitr\_AOB}$ represent the restrictions of plant diversity on AOA and AOB.

The second step of autotrophic nitrification involves the conversion of NO_2_^-^ to NO_3_^-^driven by nitrite-oxidizing bacteria [14]. The rate of NO_2_^-^ oxidation is primarily determined by the NO_2_^-^ concentration, soil temperature, soil moisture content, soil pH, and plant diversity. The calculated rate of NO_2_^-^ oxidation is obtained as follows:

$R_{Nitr\_NOB}=K_{max\_Nitr\_NOB}\times\frac{c_{{NO}_{2}^{-}}}{{c_{{NO}_{2}^{-}}+K}_{{NO}_{2}^{-}\_Nitr\_NOB}}\times F_{t\_Nitr\_NOB}\times F_{WFPS\_Nitr\_NOB}\times F_{pH\_Nitr\_NOB}\times F_{PD\_Nitr\_NOB} (19)$

Where $K_{max\_Nitr\_NOB}$ is the maximum NO_2_^-^ oxidation rates by NOB (g N m^-3^ h^-1^) (Table S5 and S7); $c_{{NO}_{2}^{-}}$ is the concentration of NO_2_^-^ in soil (mg kg^-1^); $K_{{NO}_{2}^{-}\_Nitr\_NOB}$ (4.0 g N m^-3^ h^-1^) is the half-saturation coefficients of NO_2_^-^ for NOB; $F_{t\_Nitr\_NOB}$ represent the restriction of soil temperature on NOB; $F_{WFPS\_Nitr\_NOB}$ represent the restriction of soil moisture on NOB; $F_{pH\_Nitr\_NOB}$ represent the restrictions of soil pH on NOB; $F_{PD\_Nitr\_NOB}$represent the restrictions of plant diversity on NOB.

The N_2_O production rates from autotrophic nitrification are defined as fractions of NH_4_^+^ oxidation rates:

$$\begin{aligned} {N_{2}O}_{AOA}={R_{Nitr\_AOA}\times F}_{N_{2}O\_AOA}\#\left( 20 \right) \end{aligned}$$

$$\begin{aligned} {N_{2}O}_{AOB}={R_{Nitr\_AOB}\times F}_{N_{2}O\_AOB}\#\left( 21 \right) \end{aligned}$$

where $F_{N_{2}O\_AOA}$ (0.1) and $F_{N_{2}O\_AOB}$ (0.2) are the N_2_O products ratio from NH_4_^+^ oxidation by AOA and AOB, respectively.

**(2) Heterotrophic nitrification**

Heterotrophic nitrification is a nitrification process driven by microorganisms in aerobic, acidic soils, with organic nitrogen being the primary substrate for this process [15,16]. The rate of heterotrophic nitrification is mainly influenced by soil soluble organic nitrogen, soil carbon-to-nitrogen ratio, soil temperature, soil moisture content, soil pH [17], and plant diversity. The calculated rate of heterotrophic nitrification, denoted as $R_{NitrH}$, is determined as follows:

$R_{NitrH}=K_{max\_NitrH}\times\frac{c_{DON}}{{c_{DON}+K}_{DON\_NitrH}}\times F_{t\_NitrH}\times F_{WFPS\_NitrH}\times F_{CN\_NitrH}\times F_{PD\_NitrH} (22)$

Where $K_{max\_NitrH}$ is the maximum heterotrophic nitrification rates (g N m^-3^ h^-1^) (Table S5 and S7); $c_{DON}$ is the concentration of DON in soil (mg kg^-1^); $K_{DON\_NitrH}$ (10.0 g N m^-3^ h^-1^) is the half-saturation coefficients of DON; $F_{t\_NitrH}$, $F_{WFPS\_NitrH}$, $F_{CN\_NitrH}$, $F_{PD\_Nitr\_NOB}$ represent the restriction of soil temperature, soil moisture, soil pH, soil C/N, plant diversity on heterotrophic nitrification.

N_2_O production rate from heterotrophic nitrification is proportional to the heterotrophic nitrification rate:

$$\begin{aligned} {N_{2}O}_{NitrH}={R_{NitrH}\times F}_{N_{2}O_{NitrH}}\#\left( 23 \right) \end{aligned}$$

where ${N_{2}O}_{NitrH}$ (g N m^-3^ h^-1^) is the N_2_O production rate from heterotrophic nitrification; $F_{N_{2}O\_NitrH}$(0.2) is the N_2_O production fraction during heterotrophic nitrification.

**(3) Denitrifier denitrification**

The denitrification process is a reduction process mediated by microorganisms under anaerobic conditions, where NO_3_^-^ is sequentially reduced to NO_2_^-^, NO, N_2_O or N_2_ [18]. The rate of each process depends on the concentration of nitrogen oxides (NO_3_^-^, NO_2_^-^, NO, N_2_O, N_2_) [19], soil temperature, soil moisture content, soil pH, and plant diversity. The reaction rate formula for denitrification is determined as:

$R_{NO_{X}\_Denitr}=K_{max\_NO_{X}\_Denitr}\times\frac{c_{{NO}_{X}}}{{c_{{NO}_{X}}+K}_{NO_{X}\_Denitr}}\times F_{t\_NO_{X}\_Denitr}\times F_{WFPS\_NO_{X}\_Denitr}\times F_{pH\_NO_{X}\_Denitr}\times F_{PD\_NO_{X}\_Denitr} (24)$

Where $K_{max\_NO_{X}\_Denitr}$ is the maximum reduction rates by denitrifiers (g N m^-3^ h^-1^) (Table S5 and S7); $c_{{NO}_{X}}$ is the concentration of NO_2_^-^ in soil (mg kg^-1^); $K_{NO_{X}\_Denitr}$is the half-saturation coefficients of NO_X_ ($K_{NO_{3}^{-}\_Denitr}$=3.0 g N m^-3^ h^-1^; $K_{N_{2}O\_Denitr}$=0.005 g N m^-3^ h^-1^); $F_{t\_NO_{X}\_Denitr}$, $F_{WFPS\_NO_{X}\_Denitr}$, $F_{pH\_NO_{X}\_Denitr}$, $F_{PD\_NO_{X}\_Denitr}$ represent the effects of soil temperature, soil moisture, soil pH, plant diversity on denitrification.

**(4) Nitrifier denitrification**

The denitrification process of nitrifying bacteria mainly involves AOB sequentially reducing NO_2_^-^ to NO, N_2_O, or N_2_ [20,21]. Soil moisture is a key factor controlling the denitrification process of nitrifying bacteria. The reaction rate formula for the denitrification process of nitrifying bacteria is:

$R_{NO_{X}\_AOB}=K_{max\_NO_{X}\_AOB}\times\frac{c_{{NO}_{X}}}{{c_{{NO}_{X}}+K}_{NO_{X}\_AOB}}\times F_{t\_NO_{X}\_AOB}\times F_{WFPS\_NO_{X}\_AOB}\times F_{pH\_NO_{X}\_AOB}\times F_{PD\_NO_{X}\_AOB} (25)$

Where $K_{max\_NO_{X}\_AOB}$ is the maximum reduction rates by AOB (g N m^-3^ h^-1^) (Table S5 and S7); $c_{{NO}_{X}}$ is the concentration of NO_X_ in soil (mg kg^-1^); $K_{NO_{X}\_AOB}$is the half-saturation coefficients of dissolved NO_X_ ($K_{NO_{2}^{-}\_AOB}$=0.14 g N m^-3^ h^-1^; $K_{N_{2}O\_AOB}$=0.05 g N m^-3^ h^-1^); $F_{t\_NO_{X}\_AOB}$, $F_{WFPS\_NO_{X}\_AOB}$, $F_{pH\_NO_{X}\_AOB}$, $F_{PD\_NO_{X}\_AOB}$represent the effects of soil temperature, soil moisture, soil pH, plant diversity on NO_X_ reduction by AOB.

In the MicN-SR model, five sub-processes produce N_2_O and two sub-processes consume N_2_O. Therefore, the concentration of N_2_O in soil is calculated as:

$N_{2}O={N_{2}O}_{AOA}+{N_{2}O}_{AOB}+{N_{2}O}_{NitrH}+R_{NO_{3}^{-}\_Denitr}+R_{NO_{2}^{-}\_AOB}-R_{N_{2}O\_Denitr}-R_{N_{2}O\_AOB} (26)$

Table S1 driving data used to simulate global forest soil emissions from 2019 to 2020

| **Driver** | **Data** |
| --- | --- |
| Soil temperature | CRU TSv4.05 [22] |
| Soil moisture | GLEAMv3.6b [23] |
| Soil pH | [24] |
| Soil total C | [24] |
| Soil total N | [24] |
| Soil C/N | Soil total C/ Soil total N |
| Soil NH_4_^+^ | CMIP6 [25] |
| Soil NO_3_^-^ | CMIP6 [25] |
| Vegetation mask | GLOBCOVER 2009 |

Table S2 Equations for modeling the factors affecting the N_2_O production process

|  | **Equation** | **Reference** |
| --- | --- | --- |
| Autotrophic nitrification | | |
| (1) | $F_{t\_Nitr\_AOA}={(\frac{60-Ts}{60-38})}^{3}\times e^{3\times\frac{Ts-38}{60-38}}$ | [11] |
|  | $F_{WFPS\_Nitr\_AOA}=0.2+0.8\times{(\frac{\frac{WFPS}{100}-1.05}{0.5-1.05})}^{3.5\times(\frac{1.05-0.5}{0.5-0.0012})}\times{(\frac{\frac{WFPS}{100}-0.0012}{0.5-0.0012})}^{3.5}$ | [12] |
|  | $F_{pH\_Nitr\_AOA}=0.5+0.5\times e^{-\frac{{(pH-6.0)}^{2}}{5}}$ | [13,26,27] |
|  | $\boldsymbol{F}_{\boldsymbol{PD\_Nitr\_AOA}}\mathbf{=-0.1608}\boldsymbol{\times}\mathbf{S}\mathbf{+1.1021}$ |  |
| (2) | $F_{t\_Nitr\_AOB}={(\frac{60-Ts}{60-30})}^{4}\times e^{4\times\frac{Ts-30}{60-30}}$ | [11] |
|  | $F_{WFPS\_Nitr\_AOB}=F_{WFPS\_Nitr\_AOA}$ | [12] |
|  | $F_{pH\_Nitr\_AOB}={0.4+0.6\times e}^{-\frac{{(pH-7.0)}^{2}}{4}}$ | [13,27] |
|  | $\boldsymbol{F}_{\boldsymbol{PD\_Nitr\_AOB}}\mathbf{=-0.1761}\boldsymbol{\times}\mathbf{S}\mathbf{+1.2}$ |  |
| (3) | $F_{t\_Nitr\_NOB}={(\frac{60-T}{60-33})}^{5}\times e^{5\times\frac{T-33}{60-33}}$ | [28] |
|  | $F_{WFPS\_Nitr\_NOB}={(\frac{\frac{WFPS}{100}-1.05}{0.4-1.05})}^{4.0\times(\frac{1.05-0.4}{0.4-0.0012})}\times\frac{\frac{WFPS}{100}-0.0012}{0.4-0.0012}$ | [29] |
|  | $F_{pH\_Nitr\_NOB}=0.45+0.55\times e^{-\frac{{(pH-6.5)}^{2}}{4.5}}$ | [14] |
|  | $\boldsymbol{F}_{\boldsymbol{PD\_Nitr\_NOB}}\boldsymbol{=-0.00095\times}\mathbf{S}^{\boldsymbol{2}}\boldsymbol{+0.0221\times}\mathbf{S}\boldsymbol{+1.146}$ |  |
| Heterotrophic nitrification | | |
| (4) | $F_{t\_NitrH}=max(-0.0019{Ts}^{2}+0.0943Ts-0.4617, 0)$ | [30] |
|  | $F_{WFPS\_NitrH}=max(-4.60\times({WFPS/100)}^{2}+5.51\times(\frac{WFPS}{100})-0.72, 0)$ | [30] |
|  | $F_{CN\_NitrH}=0.05\times Rcn-0.37$ | [31] |
|  | $\boldsymbol{F}_{\boldsymbol{PD\_NitrH}}\boldsymbol{=-0.0632\times}\mathbf{S}^{\boldsymbol{2}}\boldsymbol{+0.4018\times}\mathbf{S+1.0133}$ |  |
| Denitrifier denitrification | | |
| (5) | $F_{t\_{NO}_{3}^{-}\_Denitr}=e^{-\frac{{(Ts-37)}^{2}}{{25}^{2}}}$ | [32-34] |
|  | $F_{t\_N_{2}O\_Denitr}=e^{-\frac{{(Ts-42)}^{2}}{{27}^{2}}}$ | [32,34,35] |
|  | $F_{WFPS\_{NO}_{3}^{-}\_Denitr}=0.624+0.8\times\frac{arctan(0.45\pi(0.1WFPS-8.0))}{2.85}$ | [36] |
|  | $F_{WFPS\_N_{2}O\_Denitr}=0.656+0.9\times\frac{arctan(0.4\pi(0.1WFPS-9.0))}{2.55}$ | [37-39] |
|  | $F_{pH\_{NO}_{3}^{-}\_Denitr}=1-\frac{0.6}{1+e^{(pH-5.0)/1.5}}$ | [40,41] |
|  | $F_{pH\_N_{2}O\_Denitr}=1-\frac{0.7}{1+e^{(pH-6.0)/1.5}}$ | [40-42] |
|  | $\boldsymbol{F}_{\boldsymbol{PD\_}\boldsymbol{NO}_{\mathbf{3}}^{\mathbf{-}}\boldsymbol{\_Denitr}}\boldsymbol{=-0.00003\times}\mathbf{S}^{\boldsymbol{2}}\boldsymbol{-0.0126\times}\mathbf{S+0.9896}$ |  |
|  | $\boldsymbol{F}_{\boldsymbol{PD\_}\boldsymbol{N}_{\boldsymbol{2}}\boldsymbol{O}\boldsymbol{\_Denitr}}\boldsymbol{=-0.0013}\boldsymbol{\times}\mathbf{S}^{\boldsymbol{2}}\boldsymbol{+0.0113\times}\mathbf{S+0.9786}$ |  |
| Nitrifier denitrification | | |
| (6) | $F_{WFPS\_{NO}_{2}^{-}\_AOB}=0.624+0.8\times\frac{arctan(0.3\pi(0.1WFPS-7.5))}{2.85}$ | [37,39] |
|  | $F_{WFPS\_N2O\_AOB}=0.565+0.9\times\frac{arctan(0.4\pi(0.1WFPS-8.0))}{2.55}$ | [37,39] |
|  | $F_{t\_NO_{X}\_AOB}=F_{t\_Nitr\_AOB}$ | [20,21] |
|  | $F_{pH\_NO_{X}\_AOB}=F_{pH\_Nitr\_AOB}$ | [20,21] |
|  | $\boldsymbol{F}_{\boldsymbol{PD\_}\boldsymbol{NO}_{\mathbf{2}}^{\mathbf{-}}\boldsymbol{\_AOB}}\boldsymbol{=}\boldsymbol{-0.0151}\boldsymbol{\times}\mathbf{S}^{\boldsymbol{2}}\boldsymbol{+0.0527\times}\mathbf{S+0.9412}$ |  |
|  | $\boldsymbol{F}_{\boldsymbol{PD\_}\boldsymbol{N}_{\boldsymbol{2}}\boldsymbol{O}\boldsymbol{\_AOB}}\boldsymbol{=0.0099\times}\mathbf{S}^{\boldsymbol{2}}\boldsymbol{-0.0375\times}\mathbf{S+1.0135}$ |  |

where Ts is the soil temperature (℃); WFPS is the soil water-filled pore space (%); pH is the soil pH; Rcn is the soil C/N ratio; S is transformation of the number of tree species; AOA represents ammonia oxidizing archaea; AOB represents ammonia oxidizing bacteria; NOB represents nitrite oxidizing bacteria, plant diversity functions are in bold.

Table S3 Sites scale model calibration parameter information

| Processes | Parameter | Prior values | | Posterior values | Standard deviation | Definition |
| --- | --- | --- | --- | --- | --- | --- |
|  |  | Min | Max |  |  |  |
| Autotrophic nitrification | $K_{max\_Nitr\_AOA}$ | 0.000001 | 0.000015 | 0.00001115 | 0.00000431 | Maximum NH_4_^+^ oxidation rate by AOA under 1 tree species |
|  | $K_{max\_Nitr\_AOB}$ | 0.000002 | 0.0002 | 0.00006779 | 0.00000419 | Maximum NH_4_^+^ oxidation rate by AOB under 1 tree species |
|  | $K_{max\_Nitr\_NOB}$ | 0.0001 | 0.003 | 0.00136951 | 0.00097057 | Maximum NO_2_^-^ oxidation rate by NOB under 1 tree species |
| Heterotrophic nitrification | $K_{max\_NitrH}$ | 0.000005 | 0.00002 | 0.00000894 | 0.00000465 | Maximum heterotrophic nitrification rate under 1 tree species |
| Denitrifier denitrification | $K_{max\_NO_{3}^{-}\_Denitr}$ | 0.000001 | 0.00005 | 0.00004756 | 0.00000873 | Maximum NO_3_^−^ reduction rate by denitrifiers under 1 tree species |
|  | $K_{max\_N_{2}O\_Denitr}$ | 0.00002 | 0.001 | 0.00048488 | 0.00033285 | Maximum N_2_O reduction rate by denitrifiers under 1 tree species |
| Nitrifier denitrification | $K_{max\_NO_{2}^{-}\_AOB}$ | 0.0001 | 0.003 | 0.00176989 | 0.00090964 | Maximum NO_2_^-^ reduction rate by AOB under 1 tree species |
|  | $K_{max\_N_{2}O\_AOB}$ | 0.0001 | 0.003 | 0.00149544 | 0.00094952 | Maximum N_2_O reduction rate by AOB under 1 tree species |

Table S4 Global scale model calibration parameter information

| Processes | Parameter | Prior values | | Posterior values | Standard deviation | Definition |
| --- | --- | --- | --- | --- | --- | --- |
|  |  | Min | Max |  |  |  |
| Autotrophic nitrification | $K_{max\_Nitr\_AOA}$ | 0.000001 | 0.000015 | 0.00001213 | 0.00000427 | Maximum NH_4_^+^ oxidation rate by AOA under 1 tree species |
|  | $K_{max\_Nitr\_AOB}$ | 0.000002 | 0.0002 | 0.00006521 | 0.00000858 | Maximum NH_4_^+^ oxidation rate by AOB under 1 tree species |
|  | $K_{max\_Nitr\_NOB}$ | 0.0001 | 0.003 | 0.00168183 | 0.00101898 | Maximum NO_2_^-^ oxidation rate by NOB under 1 tree species |
| Heterotrophic nitrification | $K_{max\_NitrH}$ | 0.000005 | 0.00002 | 0.00000767 | 0.00000482 | Maximum heterotrophic nitrification rate under 1 tree species |
| Denitrifier denitrification | $K_{max\_NO_{3}^{-}\_Denitr}$ | 0.000001 | 0.00005 | 0.00004569 | 0.00000956 | Maximum NO_3_^−^ reduction rate by denitrifiers under 1 tree species |
|  | $K_{max\_N_{2}O\_Denitr}$ | 0.00002 | 0.001 | 0.00055647 | 0.00036351 | Maximum N_2_O reduction rate by denitrifiers under 1 tree species |
| Nitrifier denitrification | $K_{max\_NO_{2}^{-}\_AOB}$ | 0.0001 | 0.003 | 0.00139877 | 0.00103942 | Maximum NO_2_^-^ reduction rate by AOB under 1 tree species |
|  | $K_{max\_N_{2}O\_AOB}$ | 0.0001 | 0.003 | 0.00137794 | 0.00104235 | Maximum N_2_O reduction rate by AOB under 1 tree species |

**References:**

1. Bruelheide H, Nadrowski K, Assmann T *et al*. Designing forest biodiversity experiments: general considerations illustrated by a new large experiment in subtropical China. *Methods Ecol Evol* 2014; **5**: 74–89.

2. Toyoda S, Mutobe H, Yamagishi H *et al*. Fractionation of N_2_O isotopomers during production by denitrifier. *Soil Biol Biochem* 2005; **37**: 1535–1545.

3. Toyoda S, Yoshida N. Determination of Nitrogen Isotopomers of Nitrous Oxide on a Modified Isotope Ratio Mass Spectrometer. *Anal Chem* 1999; **71**: 4711-4718.

4. Lewicka-Szczebak D, Augustin J, Giesemann A *et al*. Quantifying N_2_O reduction to N_2_ based on N_2_O isotopocules-validation with independent methods (helium incubation and ^15^N gas flux method). *Biogeosciences* 2017; **14**: 711–732.

5. Toyoda S, Yano M, Nishimura S *et al*. Characterization and production and consumption processes of N_2_O emitted from temperate agricultural soils determined via isotopomer ratio analysis. *Glob Biogeochem Cycle* 2011; **25**: 1–17.

6. Wu D, Well R, Cárdenas LM *et al*. Quantifying N_2_O reduction to N_2_ during denitrification in soils via isotopic mapping approach: model evaluation and uncertainty analysis. *Environ Res* 2019; **179**: 108806.

7. Lyu S. Variability of δ^2^H and δ^18^O in soil water and its linkage to precipitation in an east asian monsoon subtropical forest plantation. *Water* 2021; **13**: 2930.

8. Ma M, Song C, Fang H *et al*. Development of a process-based N_2_O emission model for natural forest and grassland ecosystems. *J Adv Model Earth Syst* 2022; **14**: e2021MS002460.

9. Prosser JI, Nicol GW. Relative contributions of archaea and bacteria to aerobic ammonia oxidation in the environment. *Environ Microbiol* 2008; **10**: 2931–2941.

10. Li Z, Tang Z, Song Z *et al*. Variations and controlling factors of soil denitrification rate. *Glob Change Biol* 2022; **28**: 2133–2145.

11. Ouyang Y, Norton JM, Stark JM. Ammonium availability and temperature control contributions of ammonia oxidizing bacteria and archaea to nitrification in an agricultural soil. *Soil Biol Biochem* 2017; **113**: 161–172.

12. Gleeson DB, Müller C, Banerjee S *et al*. Response of ammonia oxidizing archaea and bacteria to changing water filled pore space. *Soil Biol Biochem* 2010; **42**: 1888–1891.

13. Hu H, Zhang L, Yuan C *et al*. Contrasting *Euryarchaeota* communities between upland and paddy soils exhibited similar pH-impacted biogeographic patterns. *Soil Biol Biochem* 2013; **64**: 18–27.

14. Daims H, Lücker S, Wagner M. A new perspective on microbes formerly known as nitrite-oxidizing bacteria. *Trends Microbiol* 2016; **24**: 699–712.

15. Islam A, Chen D, White RE. Heterotrophic and autotrophic nitrification in two acid pasture soils. *Soil Biol Biochem* 2007; **39**: 972–975.

16. Huygens D, Boeckx P, Templer P *et al*. Mechanisms for retention of bioavailable nitrogen in volcanic rainforest soils. *Nat Geosci* 2008; **1**: 543–548.

17. Zhang L, Zeng G, Zhang J *et al*. Response of denitrifying genes coding for nitrite (*nirK* or *nirS*) and nitrous oxide (*nosZ*) reductases to different physico-chemical parameters during agricultural waste composting. *Appl Microbiol Biotechnol* 2015; **99**: 4059–4070.

18. Norman J, Jansson PE, Farahbakhshazad N *et al*. Simulation of NO and N_2_O emissions from a spruce forest during a freeze/thaw event using an N-flux submodel from the PnET-N-DNDC model integrated to CoupModel. *Ecol Model* 2008; **216**: 18–30.

19. Del Grosso SJ, Parton WJ, Mosier AR *et al*. General model for N_2_O and N_2_ gas emissions from soils due to dentrification. *Glob Biogeochem Cycle* 2000; **14**: 1045–1060.

20. Shaw LJ, Nicol GW, Smith Z *et al*. *Nitrosospira* spp. can produce nitrous oxide via a nitrifier denitrification pathway. *Environ Microbiol* 2006; **8**: 214–222.

21. Venterea RT. Nitrite-driven nitrous oxide production under aerobic soil conditions: kinetics and biochemical controls. *Glob Change Biol* 2007; **13**: 1798–1809.

22. Harris I, Osborn TJ, Jones P *et al*. Version 4 of the CRU TS monthly high-resolution gridded multivariate climate dataset. *Sci Data* 2020; **7**: 1–18.

23. Martens B, Miralles DG, Lievens H *et al*. GLEAM v3: satellite-based land evaporation and root-zone soil moisture. *Geosci Model Dev* 2017; **10**: 1903–1925.

24. Shangguan W, Dai Y, Duan Q *et al*. A global soil data set for earth system modeling. *J Adv Model Earth Syst* 2014; **6**: 249–263.

25. Bentsen M, Oliviè DJL, Seland Ø *et al*. Data from: NCC NorESM2-MM model output prepared for CMIP6 ScenarioMIP. 2019. <https://doi.org/10.22033/ESGF/CMIP6.608>

26. Kim DG, Vargas R, Bond-Lamberty B *et al*. Effects of soil rewetting and thawing on soil gas fluxes: a review of current literature and suggestions for future research. *Biogeosciences* 2012; **9**: 2459–2483.

27. Tzanakakis VA, Taylor AE, Bakken LR *et al*. Relative activity of ammonia oxidizing archaea and bacteria determine nitrification-dependent N_2_O emissions in Oregon forest soils. *Soil Biol Biochem* 2019; **139**: 1–6.

28. Blackburne R, Yuan Z, Keller J. Partial nitrification to nitrite using low dissolved oxygen concentration as the main selection factor. *Biodegradation* 2008; **19**: 303–312.

29. Taylor AE, Myrold DD, Bottomley PJ. Temperature affects the kinetics of nitrite oxidation and nitrification coupling in four agricultural soils. *Soil Biol Biochem* 2019; **136**: 1–9.

30. Zhang Y, Ma M, Fang H *et al*. Impacts of nitrogen addition on nitrous oxide emission: comparison of five nitrous oxide modules or algorithms. *Ecol Model* 2020; **421**: 1–8.

31. Zhang J, Müller C, Cai Z. Heterotrophic nitrification of organic N and its contribution to nitrous oxide emissions in soils. *Soil Biol Biochem* 2015; **84**: 199–209.

32. Benoit M, Garnier J, Billen G. Temperature dependence of nitrous oxide production of a luvisolic soil in batch experiments. *Process Biochem* 2015; **50**: 79–85.

33. Li P, Wang Y, Zuo J *et al*. Nitrogen removal and N_2_O accumulation during hydrogenotrophic denitrification: influence of environmental factors and microbial community characteristics. *Environ Sci Technol* 2017; **51**: 870–879.

34. Wang Y, Wang H, Wang Z-L *et al*. Effect of litter layer on soil–atmosphere N2O flux of a subtropical pine plantation in China. *Atmos Environ* 2014; **82**: 106-112.

35. Qin S, Yuan H, Hu C *et al*. Determination of potential N_2_O-reductase activity in soil. *Soil Biol Biochem* 2014; **70**: 205–210.

36. Bao Q, Ju X, Gao B *et al*. Response of nitrous oxide and corresponding bacteria to managements in an agricultural soil. *Soil Sci Soc Am J* 2012; **76**: 130–141.

37. Kool DM, Dolfing J, Wrage N *et al*. Nitrifier denitrification as a distinct and significant source of nitrous oxide from soil. *Soil Biol Biochem* 2011; **43**: 174–178.

38. Morley N, Baggs EM, Dörsch P *et al*. Production of NO, N_2_O and N_2_ by extracted soil bacteria, regulation by NO_2_^-^ and O_2_ concentrations. *FEMS Microbiol Ecol* 2008; **65**: 102–112.

39. Zhu X, Burger M, Doane TA *et al*. Ammonia oxidation pathways and nitrifier denitrification are significant sources of N_2_O and NO under low oxygen availability. *Proc Natl Acad Sci USA* 2013; **110**: 6328–6333.

40. Bergaust L, Mao Y, Bakken LR *et al*. Denitrification response patterns during the transition to anoxic respiration and posttranscriptional effects of suboptimal pH on nitrous oxide reductase in *Paracoccus denitrificans*. *Appl Environ Microbiol* 2010; **76**: 6387–6396.

41. Lim NYN, Frostegard A, Bakken LR. Nitrite kinetics during anoxia: the role of abiotic reactions versus microbial reduction. *Soil Biol Biochem* 2018; **119**: 203–209.

42. Hénault C, Bourennane H, Ayzac A *et al*. Management of soil pH promotes nitrous oxide reduction and thus mitigates soil emissions of this greenhouse gas. *Sci Rep* 2019; **9**: 1–11.
