# Supplementary material for: Enhanced plant diversity reduces nitrous oxide emissions in forest soils worldwide
Source: Natl Sci Rev. 2025 May 13;12(7):nwaf186. doi: 10.1093/nsr/nwaf186 (PMC12168753; doi:10.1093/nsr/nwaf186)
Supplement: nwaf186_Supplemental_File [file nwaf186_supplemental_file.zip › 1. Supplementary tables and figures.docx]

**Supplementary tables and figures**

**Enhanced plant diversity reduces nitrous oxide emissions in forest soils worldwide**

Table S1 Species composition of plots at different levels of tree diversity

| **Tree diversity** | **Species composition** | | | | | | | |
| --- | --- | --- | --- | --- | --- | --- | --- | --- |
| 1 species | LiGl | ScSu | CaEy | CaSc | CyMy | CyGl | QuFa | CaHe |
|  | QuSe | KoBi | LiFo | SaMu | SaSe | RhCh | ChAx | NySi |
| 2 species | CaEy–CyMy | | CaHe–NySi | | LiFo–SaMu | | ChAx–SaSe | |
|  | LiGl–KoBi | | ScSu–RhCh | | CaSc–QuSe | | CyGl–QuFa | |
| 4 species | CaHe–LiFo–NySi–SaMu | | | | CyGl–ScSu–QuFa–RhCh | | | |
|  | CaSc–ChAx–QuSe–SaSe | | | | CaEy–CyMy–LiGl–KoBi | | | |
| 8 species | QuFa–CyGl–ScSu–RhCh–CyMy–CaEy–KoBi–LiGl | | | | | | | |
|  | CaHe–NySi–LiFo–SaMu–ChAx–SaSe–QuSe–CaSc | | | | | | | |
| 16 species | QuFa–CyGl–ScSu–RhCh–CyMy–CaEy–KoBi–LiGl–CaHe–NySi–LiFo–SaMu–ChAx–SaSe–QuSe–CaSc | | | | | | | |
| 24 species | QuFa–CyGl–ScSu–RhCh–CyMy–CaEy–KoBi–LiGl–CaHe–NySi–LiFo–SaMu–ChAx–SaSe–QuSe–CaSc–CaCa–AcDa–CiCa–DaOl–DiGl–MeAz–QuAc–SaDi | | | | | | | |

AcDa (*Acer davidii*), CaCa (*Castanopsis carlesii*), CaEy (*Castanopsis eyrei*) CaHe (*Castanea henryi*), CaSc (*Castanopsis sclerophylla*), ChAx (*Choerospondias axillaris*), CiCa (*Cinnamomum camphora*), CyGl (*Cyclobalanopsis glauca*), CyMy (*Cyclobalanopsis myrsinifolia*), DaOl (*Daphniphyllum oldhamii*), DiGl (*Diospyros glaucifolia*), KoBi (*Koelreuteria bipinnata*), LiFo (*Liquidambar formosana*), LiGl (*Lithocarpus glaber*), MeAz (*Melia azedarach*), NySi (*Nyssa sinensis*), QuAc (*Quercus acutissima*), QuFa (*Quercus fagri*), QuSe (*Quercus serrata*), RhCh (*Rhus chinensis*), SaDi (*Sapium discolor*) SaMu (*Sapindus mukorossi*), SaSe (*Sapium sebiferum*), ScSu (*Schima superba*).

Table S2 Partial correlation analysis between N_2_O fluxes and tree diversity

| **Primary Variable X** | **Primary Variable Y** | **Control Variable (s)** | **Partial Correlation Coefficient (*r*)** | **t value** | ***P* value** |
| --- | --- | --- | --- | --- | --- |
| N_2_O fluxes | Plant species richness | Soil moisture | -0.16 | -4.54 | <0.001 |
| N_2_O fluxes | Plant species richness | Soil temperature | -0.16 | -4.62 | <0.001 |
| N_2_O fluxes | Plant species richness | Above-ground biomass | -0.24 | -7.59 | <0.001 |
| N_2_O fluxes | Plant species richness | Soil organic carbon | -0.16 | -5.06 | <0.001 |
| N_2_O fluxes | Plant species richness | Soil NH_4_^+^-N | -0.19 | -5.9 | <0.001 |
| N_2_O fluxes | Plant species richness | Soil NO_3_^-^-N | -0.14 | -4.18 | <0.001 |

Table S3 Model parameters used in the SP/O mapping model

| **Pathway** | **Literature values^a^** | | **Substrate isotope values^b^** | **Substrate-corrected values** |
| --- | --- | --- | --- | --- |
|  | **δ^15^N^SP^_N2O_** | **δ^18^O_N2O_** | **δ^18^O_H2O_** | **δ^18^O_N2O_** |
| bD | **-1.9 ± 4.6** | 19.0 ± 2.1 | -6.23 ± 1.05 | **12.77 ± 2.1** |
| nD | **-5.9 ± 6.5** | 15.7 ± 2.9 | -6.23 ± 1.05 | **9.47 ± 2.9** |
| fD | **33.6 ± 2.5** | 46.9 ± 3.8 | -6.23 ± 1.05 | **40.67 ± 3.8** |
| Ni | **34.6 ± 2.9** | 23.5 ± 2.1 | -6.23 ± 1.05 | **17.27 ± 2.1** |
| Reduction | **-6.0 ± 1.4** | -15.9 ± 4.7 | -6.23 ± 1.05 | **-22.13 ± 4.7** |

The values are the median ± SE for measured values; ^a^the literature values are from Lewicka-Szczebak *et al*. (2020), in which the values were summarized via literature retrieval. ^b^the literature values are from Lyu *et al*. (2021). The final input values for the SP/O model are in bold.

Fig. S1 Schematic diagram showing the spatial distribution and tree diversity levels in BEF-China (modified from Ma, 2013).

Fig. S2 Subgroup analyses of the effect sizes of tree diversity on N_2_O fluxes from the literature dataset (a) and from the present study (BEF-China experiment) (b). The black points in the figure represent the effect sizes of species mixtures on N_2_O fluxes, while the black error bars indicate the 95% confidence intervals. The blue points represent the effect sizes of different tree diversity on N_2_O fluxes, and the purple points represent the effect sizes of total species mixtures on N_2_O fluxes; significance levels at *P*<0.05, *P*<0.01, *P*<0.001 indicated by *, **, ***, respectively.

Fig. S3 Effects of tree diversity on soil N_2_O fluxes during the growing and non-growing seasons.

Fig. S4 Changes in tree diversity on soil N_2_O isotope signature values (δ^15^N^SP^_N2O_、δ^18^O_N2O_ and δ^15^N^bulk^_N2O_) (a) and isotope mapping model (SP/O MAP) traces N_2_O production pathway (b).

Fig. S5 Pearson’s correlation coefficients for relations between tree diversity, soil N_2_O fluxes, nitrification N_2_O production, denitrification N_2_O production, denitrification N_2_O reduction and soil physicochemical properties. The black crosses indicate non-significant correlations.

Fig. S6 The effects of tree diversity on soil moisture (a) and soil temperature (b), as well as the differences in soil moisture (c) and soil temperature (d) between growing and non-growing seasons. The black points in the center of each box represent the mean values for soil moisture and soil temperature. The boxes display the 25th, median, and 75th percentiles, while the whiskers extending from the boxes indicate the standard deviation. The colored regions represent the distribution of the soil moisture and soil temperature data. Significance levels at *P*<0.05, *P*<0.01, *P*<0.001 indicated by *, **, ***, respectively.

Fig. S7 Validation of MicN-SR and MicN at global forest. Red represents MicN-SR model and blue represents MicN model.

Fig. S8 MicN and MicN-SR models simulate annual average soil N_2_O emissions from tropical/subtropical and temperate/boreal forests.
